# Risk of mortality between warfarin and direct oral anticoagulants: population-based cohort studies

**DOI:** 10.1186/s12916-024-03808-y

**Published:** 2024-12-23

**Authors:** Zixuan Wang, Julian Matthewman, John Tazare, Qiuyan Yu, Ka Shing Cheung, Celine S. L. Chui, Esther W. Y. Chan, Krishnan Bhaskaran, Liam Smeeth, Ian C. K. Wong, Ian J. Douglas, Angel Y. S. Wong

**Affiliations:** 1 Department of Non-Communicable Disease Epidemiology, Faculty of Epidemiology & Population Health, London, School of Hygiene and Tropical Medicine , London, UK; 2 Laboratory of Data Discovery for Health (D24H), Hong Kong, China; 3 School of Pharmacy, School of Health Sciences, College of Medicine and Health, University of Birmingham, Birmingham, UK; 4 Department of Medical Statistics, Faculty of Epidemiology & Population Health, London, School of Hygiene and Tropical Medicine , London, UK; 5 Department of Medicine, School of Clinical Medicine, Queen Mary Hospital, The University of Hong Kong, Hong Kong, China; 6 Department of Medicine, The University of Hong Kong-Shenzhen Hospital, Shenzhen, China; 7 School of Nursing, Li Ka Shing Faculty of Medicine, The University of Hong Kong, Hong Kong, China; 8 School of Public Health, Li Ka Shing, Faculty of Medicine, The University of Hong Kong, Hong Kong, China; 9 Centre for Safe Medication Practice and Research, Department of Pharmacology and Pharmacy, The University of Hong Kong, Hong Kong, China; 10 Aston School of Pharmacy, Aston University, Birmingham, UK

**Keywords:** Warfarin, Direct anticoagulant, Mortality

## Abstract

**Background:**

Direct oral anticoagulants (DOACs) have been reported to be associated with a higher risk of mortality compared with an older alternative, warfarin using primary care data in the United Kingdom (UK). However, other studies observed contradictory findings. We therefore aimed to investigate the association between mortality and warfarin, compared with DOACs.

**Methods:**

We conducted cohort studies using UK Clinical Practice Research Datalink (CPRD) Aurum and Hong Kong Clinical Data Analysis and Reporting System (CDARS) to identify the association between warfarin and hazard of mortality, compared to DOACs. Individuals with non-valvular atrial fibrillation aged ≥ 18 years who had first anticoagulant therapy (warfarin or DOAC) during 1/1/2011–31/12/2019 were included.

**Results:**

Compared with DOAC use, a lower hazard of all-cause mortality was found in warfarin users (hazard ratio (HR) = 0.81, 95% confidence interval (CI) = 0.77–0.86) in CPRD; while a higher hazard was observed in warfarin users (HR = 1.31, 95% CI = 1.24–1.39) in CDARS, versus DOAC users. In our exploratory analysis, consistent results were seen in both databases when stratified warfarin users by time in therapeutic range (TTR) using post-baseline measurements: a lower hazard of all-cause mortality in warfarin users with TTR ≥ 65% (CPRD: HR = 0.68, 95% CI = 0.65–0.72; CDARS: HR = 0.86, 95% CI = 0.77–0.96) and increased hazard in warfarin users with TTR < 65% (CPRD: HR = 1.14, 95% CI = 1.05–1.23; CDARS: HR = 1.59, 95% CI = 1.50–1.69), versus DOAC users.

**Conclusions:**

The differences in hazard of all-cause mortality associated with warfarin compared with DOAC, in part may depend on anticoagulation control in warfarin users. Notably, this study is unable to establish a causal relationship between warfarin and mortality stratified by TTR, versus DOACs, requiring future studies for further investigation.

**Supplementary Information:**

The online version contains supplementary material available at 10.1186/s12916-024-03808-y.

## Background

Oral anticoagulants (OACs), including warfarin and direct oral anticoagulants (DOACs) as a newer alternative, are extensively used to prevent thrombosis and reduce the risk of ischaemic stroke in patients with non-valvular atrial fibrillation [1, 2]. Increasing trends in prescribing DOACs were reported worldwide during the last decade as they have more rapid action and no requirement for international normalised ratio (INR) blood test monitoring, compared with warfarin [3–7].

Previous studies have been widely conducted to assess the risk of cardiovascular events, including ischaemic stroke, venous thromboembolism, myocardial infarction, and major bleeding, comparing the use of warfarin with DOACs [8–11]. Nevertheless, the association between oral anticoagulation therapy choice and overall mortality remains unclear. Three recent studies using routine clinical data showed a higher hazard of all-cause mortality associated with DOACs, compared with warfarin [12, 13]. However, this conflicted with findings reported in other observational studies [14–17] and a systematic review using pooled data from randomised controlled trials [18], suggesting that DOACs were associated with a lower hazard of all-cause mortality compared with warfarin. This systematic review also reported that the hazard of mortality varied across anticoagulation control measured by time in therapeutic range (TTR) for warfarin, where no difference in hazard of mortality was found in DOAC users compared with warfarin users with TTR ≥ 65% but a 15% lower hazard in DOAC users compared with warfarin users with TTR < 65% [18]. Given that the characteristics of patients enrolled in clinical trials and their adherence to medications may differ from those observed in routine clinical practice, further investigation is required to understand the role of TTR in the hazard of mortality in routine clinical settings. Importantly, whether the observed hazard of mortality varied due to cardiovascular effects through anticoagulation control was still uncertain as specific cause of mortality was not studied in the systematic review. There was also a lack of studies exploring specific causes of mortality between OAC.

Our study therefore aimed to [1] examine the association between all-cause and cause-specific mortality (death due to circulatory diseases, digestive diseases, renal and genitourinary system disease, infectious and parasitic diseases, neoplasms, respiratory diseases and other diseases) and warfarin, compared with DOACs in people with non-valvular atrial fibrillation; and [2] investigate whether the hazard of mortality may differ across the range of anticoagulation control (TTR ≥ 65% and TTR < 65%) and other subgroups, to identify people at high risk.

## Methods

### Data source, study design and study population

We conducted two cohort studies using data from England and Hong Kong during the study period (1 Jan 2011 to 31 Dec 2019) using comparable protocols.

In England, we used general practice (GP) data from the Clinical Practice Research Datalink (CPRD) Aurum with linked data on death registration (Office for National Statistics, ONS), hospital admission (Hospital Episode Statistics Admitted Patient Care, HES APC) and deprivation (patient-level and practice-level Index of Multiple Deprivation, IMD) [19–22]. In Hong Kong, we used the Clinical Data Analysis and Reporting System (CDARS), which was developed by the Hospital Authority (HA). It contains anonymised electronic health records (EHRs) of all local residents (over 7.6 million) from public hospitals and clinics in Hong Kong including data from both inpatient and outpatient settings. Data validation has demonstrated high accuracy in both databases with high-quality epidemiological studies on cardiovascular diseases and mortality [23–27]. Details of each database can be found in Additional file 1 Sect. 1.1.

We included people aged 18 years or older with a diagnosis of non-valvular atrial fibrillation who initiated their first treatment with any OAC between 1 Jan 2011 and 31 Dec 2019. The index date was defined as the date of the first recorded prescription of OACs during the study period. To ensure that we have reliable measures of drug use and baseline covariates, we required that all participants had at least 1-year continuous registration before OAC initiation (CPRD Aurum only).

We excluded people with missing data on gender or date of birth in the record, and those with end of follow-up equal to the index date. Moreover, people with a record of mitral stenosis, prosthetic mechanical valves, chronic kidney disease stage V (estimated glomerular filtration rate [eGFR] < 15 ml/min or on dialysis) or antiphospholipid antibody syndrome before study start were excluded because DOACs are not recommended for use in these patient groups. People receiving any OAC (warfarin, dabigatran, rivaroxaban, apixaban or edoxaban) before the study period were also excluded.

### Exposure and comparator, outcome, and covariates

The exposed group was those who ever received the first warfarin prescription while the comparison group was those who ever received the first DOAC prescription (dabigatran, rivaroxaban, apixaban or edoxaban) during the study period.

The primary outcome was all-cause mortality. Secondary outcomes included death due to circulatory diseases, digestive diseases, renal and genitourinary system diseases, infectious and parasitic diseases, neoplasms, respiratory diseases and other diseases respectively using International Classification of Diseases, Tenth Revision (ICD-10) codes [26].

The follow-up period commenced from the index date until outcome occurrence (i.e. death), OAC switching between warfarin and DOAC (on the day before the date when another OAC was prescribed), transferring out of the practice (CPRD Aurum only), last data collection date for the practice (CPRD Aurum only) or end of the study, whichever came first. Individuals remained in their original respective treatment groups during follow-up.

We selected and included various factors such as demographic and lifestyle characteristics, comorbidities, co-medications, and polypharmacy, as potential confounders to control for confounding based on epidemiological and clinical knowledge [28–31]. It is noted that proxies for high blood pressure, body mass index (BMI), alcohol consumption and smoking were used in CDARS (detailed in Additional file 1 Sect. 1.2).

### Statistical analysis

Analyses were conducted separately in CPRD Aurum and CDARS. Cox proportional hazards regression model was used to compute hazard ratios (HRs) with 95% confidence intervals (CIs) of the associations. Propensity score (PS, defined as the probability of treatment conditional on observed covariates [32], estimated from logistic regression) weighting using inverse probability of treatment weights (IPTW) was applied to balance characteristics in the exposed and comparison groups [33]. People with extreme PS were trimmed by excluding regions of PS nonoverlap in both groups to reduce the potential effects of residual confounding [34]. Moreover, as the proportion of missingness of lifestyle data (BMI, smoking status, and alcohol consumption status) was low in CPRD Aurum (< 7%), we used a complete-case approach in the main analysis. We also accounted for competing hazards by modelling the cause-specific hazard (i.e. censoring other deaths which was not the outcome of interest for specific causes of death). Furthermore, follow-up periods were classified as 0–1, 0–2, 0–3, 0–4, 0–5 and 0– > 5 year to investigate whether the effect was short-term or long-term. Additionally, we conducted subgroup analyses to investigate whether the hazard varied by calendar year of drug initiation, age, gender, bodyweight, polypharmacy, and renal function at baseline.

We further conducted analyses to [1] compare the hazard of outcomes in warfarin users with individual DOAC users, respectively; and [2] stratify warfarin users into two groups by TTR (TTR ≥ 65% and TTR < 65%) comparing with DOACs separately as an exploratory analysis. Sixty-five per cent was chosen as the cut-off value as TTR < 65% usually indicates poor anticoagulation control in clinical practice [35]. We used INR control measurement to be the proxy for warfarin adherence as it is poorly recorded in EHR data [36, 37]. Details of TTR calculation could be found in Additional file 1 Sect. 1.3. In addition, we conducted post hoc analyses to evaluate if the difference in hazard of mortality between warfarin (TTR ≥ 65% and TTR < 65%) and DOACs can be attributed to cardiovascular causes by including circulatory mortality and non-circulatory mortality as outcomes.

We repeated the main analysis in several sensitivity analyses and performed quantitative bias analyses shown in Table 1.

**Table 1:** Sensitivity analyses and quantitative bias analysis

**Sensitivity analysis**
1. Performing multivariable regression model
2. Addressing missing values for BMI, smoking status, and alcohol consumption status in CPRD Aurum by multiple imputation
3. Only using covariates available in both settings for PS weighting
4. Excluding those with extreme scores in the upper or lower tail of the propensity score distribution to minimise the potential impact of unmeasured covariates and prevent any bias resulting from incomplete information regarding significant hazard factors for adverse outcomes [2]. PS were recalculated after trimming. In order to determine the appropriate thresholds for trimming, we created 20 strata of 5% each for the distribution of PS (Appendix 2, Table S1)
5. Restricting study period to 2014–2019 as the growth of prescribing trend of DOACs was more stable from 2014 onwards
6. Censoring prescription discontinuation: follow-up ends when patients discontinued their treatment. We assumed continuous treatment of warfarin or DOACs if the treatment gaps between two prescriptions were ≤ 30 days. Therefore, if the subsequent treatment had a treatment gap of > 30 days with the former prescription, we defined the treatment discontinuation as the prescription end date of the former prescription + treatment gap (i.e. 30 days)
**Quantitative bias analysis**
We conducted a post hoc *E*-value calculation to determine the minimum required strength of association between an unmeasured covariate and either the exposure or the outcome, conditioned on the measured confounders, that could fully account for the observed non-null adjusted associations in CPRD Aurum

*Abbreviations*: *BMI* body mass index, *CPRD* Clinical Practice Research Datalink, *PS* propensity score, *DOACs* direct oral anticoagulants

STATA/MP 17, R 4.3.1 and SAS 9.4 were used for data processing and analyses.

## Results

**Fig. 1:**
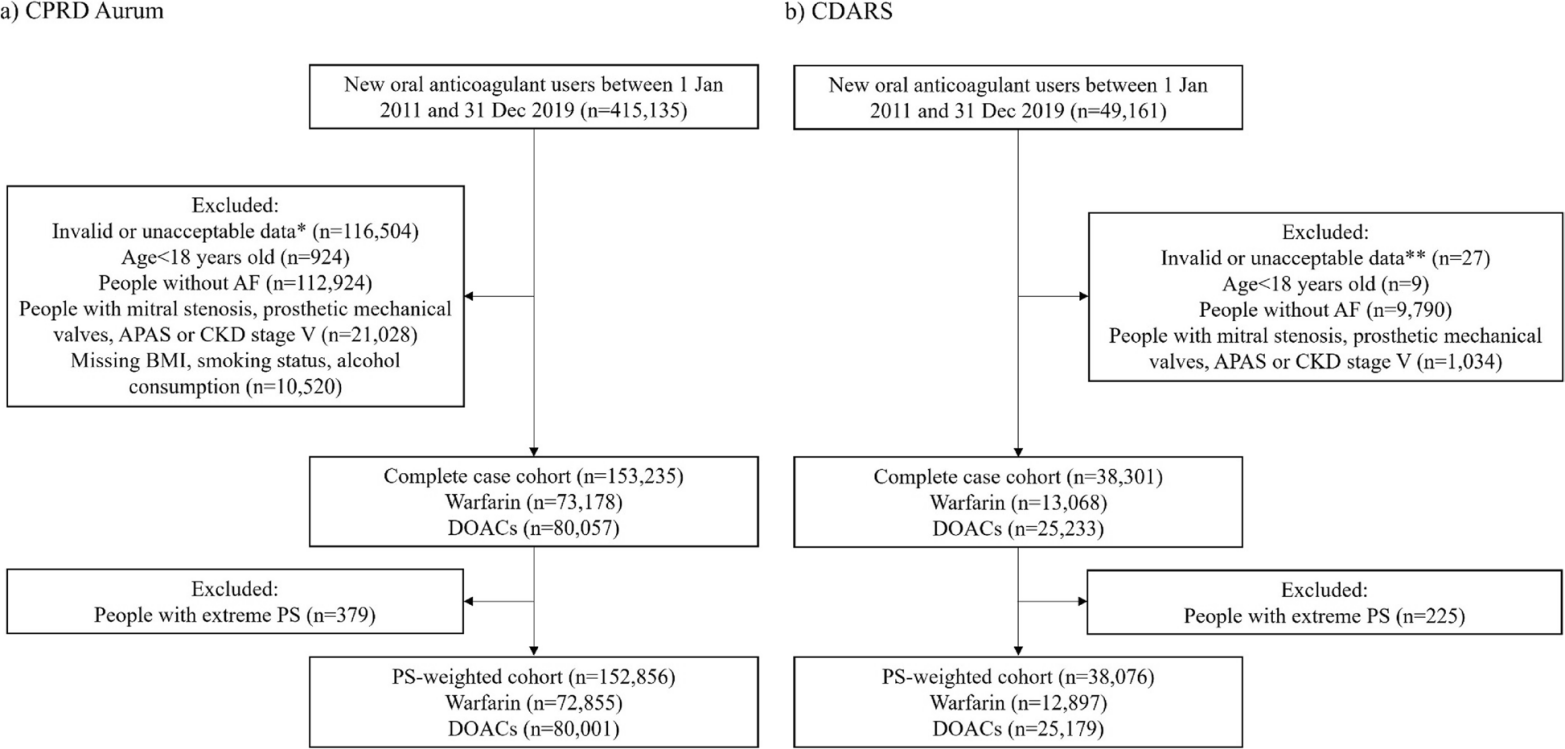
Flow chart of cohort identification in CPRD Aurum (**a**) and CDARS (**b**). Abbreviations: CPRD, Clinical Practice Research Datalink; CDARS, Clinical Data Analysis and Reporting System; DOAC, direct oral anticoagulant; BMI, body mass index; AF, atrial fibrillation; APAS, antiphospholipid antibody syndrome; CKD, chronic kidney disease; PS, propensity score. * refers to less than 1-year continuous registration, index date = end date or unacceptable data in CPRD Aurum. ** refers to missing date of birth or index date = end date in CDARS

The study flowchart, cohort baseline characteristics and details of each database are shown in Fig. 1, Table 2 and Additional file 1 Sect. 1.1. Overall, 191,536 new OAC users (153,235 in CPRD Aurum and 38,301 in CDARS) were identified, while 3048 of them (1.59%) switched treatment between OAC during follow-up (2633 in CPRD Aurum; 415 in CDARS). The median follow-up was 3.52 years (interquartile range (IQR): 1.46–5.37) in CPRD Aurum and 2.47 years (IQR: 0.98–4.61) in CDARS, respectively. Of them, 52,801 (27.57%) people (CPRD Aurum: 44,679 (29.16%); CDARS: 8122 (21.21%)) died during the study period.

**Table 2:** Selected characteristics of warfarin and DOACs users in CPRD Aurum and CDARS

	**CPRD Aurum**		**CDARS**	
	**DOAC users** *N* = 80,057	**Warfarin users** *N* = 73,178	**DOAC users** *N* = 25,233	**Warfarin users** *N* = 13,068
**Age at index date (years, median, IQR)**	77.84 (70.13, 84.44)	75.95 (68.38, 82.08)	77.18 (68.60, 83.55)	73.89 (64.26, 81.16)
**Follow-up (years, median, IQR)**	2.03 (0.91, 3.45)	5.15 (3.16, 6.81)	1.98 (0.79, 3.69)	3.87 (1.73, 6.37)
**Age group**				
18– < 40	236 (0.29)	297 (0.41)	84 (0.33)	148 (1.13)
40– < 50	1095 (1.37)	1348 (1.84)	345 (1.37)	427 (3.27)
50– < 60	4590 (5.73)	4809 (6.57)	1640 (6.50)	1510 (11.55)
60– < 70	13,797 (17.23)	15,288 (20.89)	5180 (20.53)	3117 (23.85)
70– < 80	26,805 (33.48)	27,143 (37.09)	8081 (32.03)	4076 (31.19)
80 +	33,534 (41.89)	24,293 (33.20)	9903 (39.25)	3790 (29.00)
** Gender (female)**	35,790 (44.71)	32,052 (43.80)	12,641 (50.10)	5885 (45.03)
**Calendar year at cohort entry**				
2011	65 (0.08)	12,977 (17.73)	509 (2.02)	2857 (21.86)
2012	1064 (1.33)	14,497 (19.81)	840 (3.33)	1394 (10.67)
2013	3144 (3.93)	14,276 (19.51)	1256 (4.98)	1408 (10.77)
2014	6261 (7.82)	12,705 (17.36)	1738 (6.89)	1514 (11.59)
2015	11,294 (14.11)	9263 (12.66)	2476 (9.81)	1451 (11.10)
2016	14,068 (17.57)	4852 (6.63)	3170 (12.56)	1315 (10.06)
2017	15,086 (18.84)	2407 (3.29)	4069 (16.13)	1199 (9.18)
2018	15,290 (19.10)	1292 (1.77)	4752 (18.83)	1131 (8.52)
2019	13,785 (17.22)	909 (1.24)	6423 (25.45)	817 (6.25)
** CHA2DS2 VASc score (median, IQR)**	4 (3, 5)	4 (2, 5)	3 (2, 5)	3 (2, 5)
** HASBLED score (median, IQR)**	3 (2, 4)	3 (2, 4)	2 (1, 3)	2 (1, 3)
BMI category^a^				
Underweight	1875 (2.34)	1067 (1.46)	NA	NA
Normal weight	22,622 (28.26)	18,537 (25.33)	NA	NA
Overweight or obese	55,560 (69.40)	53,574 (73.21)	NA	NA
** Overweight/obesity/other related lipid metabolism disorders**	NA	NA	5347 (21.35)	2550 (19.51)
Smoking status^a^				
Non-smoker	16,971 (21.20)	16,466 (22.50)	NA	NA
Current smoker	16,979 (21.21)	16,383 (22.39)	NA	NA
Ex-smoker	46,107 (57.59)	40,329 (55.11)	NA	NA
** Chronic obstructive pulmonary disease**	NA	NA	1932 (7.61)	998 (7.62)
Alcohol consumption status^a^				
Non-alcohol user	7062 (8.82)	6345 (8.67)	NA	NA
Current alcohol user	62,097 (77.57)	55,701 (76.12)	NA	NA
Ex-alcohol user	10,898 (13.61)	11,132 (15.21)	NA	NA
** Alcohol related disorders**	NA	NA	255 (1.00)	182 (1.39)
**Ethnicity**				
White	76,894 (96.05)	70,188 (95.91)	NA	NA
South Asian	1321 (1.65)	1298 (1.77)	NA	NA
Black	830 (1.04)	773 (1.06)	NA	NA
Other	342 (0.43)	347 (0.47)	NA	NA
Mixed	214 (0.27)	189 (0.26)	NA	NA
Not stated	303 (0.38)	216 (0.30)	NA	NA
**Index of multiple deprivation**				
Quintile 1 (least deprived)	18,771 (23.45)	17,023 (23.26)	NA	NA
Quintile 2	18,062 (22.56)	16,471 (22.51)	NA	NA
Quintile 3	15,734 (19.65)	14,603 (19.96)	NA	NA
Quintile 4	14,359 (17.94)	13,121 (17.93)	NA	NA
Quintile 5 (most deprived)	13,131 (16.40)	11,960 (16.34)	NA	NA
**Category of GP consultation within 1 year before cohort entry**				
12 + visits	66,261 (82.77)	61,778 (84.42)	NA	NA
1–11 visit(s)	13,082 (16.34)	10,162 (13.89)	NA	NA
0 visit	714 (0.89)	1238 (1.69)	NA	NA
** Polypharmacy (≥ 5 drugs) within 90 days before cohort entry**	65,693 (82.06)	59,916 (81.88)	13,221 (52.40)	8854 (67.75)
**Comorbidities at cohort entry**				
Bleeding—gastrointestinal bleeding	15,097 (18.86)	10,367 (14.17)	1925 (7.63)	1077 (8.24)
Bleeding—intracranial haemorrhage	972 (1.21)	520 (0.71)	696 (2.76)	405 (3.10)
Bleeding—other bleeding	37,414 (46.73)	28,211 (38.55)	2232 (8.85)	1170 (8.95)
Chronic renal failure	20,170 (25.19)	17,962 (24.55)	611 (2.42)	1260 (9.64)
Diabetes mellitus	21,998 (27.48)	17,853 (24.40)	7650 (30.32)	4058 (31.05)
Heart failure	18,947 (23.67)	15,545 (21.24)	5569 (22.07)	4255 (32.56)
Hypertension^a^	61,985 (77.43)	55,211 (75.45)	12,790 (50.69)	6455 (49.40)
Ischaemic heart disease	30,128 (37.63)	26,209 (35.82)	5388 (21.35)	3211 (24.57)
Ischaemic stroke or transient ischaemic attacks	16,373 (20.45)	11,290 (15.43)	5481 (21.72)	2805 (21.46)
Liver disease	2922 (3.65)	1599 (2.19)	1265 (5.01)	832 (6.37)
Peripheral artery disease	6106 (7.63)	5155 (7.04)	217 (0.86)	207 (1.58)
Venous thromboembolism	6096 (7.61)	5391 (7.37)	303 (1.20)	753 (5.76)
**Co-medications within 90 days before cohort entry**				
ACEI/ARB	40,906 (51.10)	39,890 (54.51)	12,302 (48.75)	6409 (50.96)
Antiarrhythmics	3578 (4.47)	4395 (6.01)	4070 (16.13)	2205 (16.87)
Antiplatelets	12,114 (15.13)	8820 (12.05)	2670 (10.58)	1408 (10.77)
Aspirin	26,497 (33.10)	34,453 (47.08)	15,556 (61.65)	8032 (61.46)
Beta-blockers	50,671 (63.29)	45,539 (62.23)	15,511 (61.47)	7629 (58.38)
Calcium channel blockers	27,229 (34.01)	25,694 (35.11)	14,693 (58.23)	6808 (52.10)
H2 blockers	6463 (8.07)	4151 (5.67)	11,994 (47.53)	6234 (47.70)
NSAIDs	9773 (12.21)	8470 (11.57)	1308 (5.18)	653 (5.00)
PPI	33,628 (42.01)	27,947 (38.19)	9133 (36.19)	4059 (31.06)

*Abbreviations***:** *CPRD* Clinical Practice Research Datalink, *CDARS* Clinical Data Analysis and Reporting System, *DOAC* direct oral anticoagulant, *IQR* interquartile range, *BMI* body mass index, *GP* general practice, *ACEI* Angiotensin-Converting Enzyme Inhibitors, *ARB* Angiotensin Receptor Blocker, *H2* *blocker* Histamine Type-2 Receptor Antagonists/Blockers, *NSAIDs* Non-Steroidal Anti-inflammatory Drugs, *PPI* Proton-Pump Inhibitor, *NA* not applicable

^a^Use relevant diagnoses as proxy in CDARS: BMI: diagnosis of overweight, obesity or other hyperalimentation and disorders of lipid metabolism; smoking status: chronic obstructive pulmonary disease (COPD); alcohol consumption status: alcohol related disorders; hypertension: related hypertensive disorders only

Overall, in both settings, warfarin users were more likely to be younger and current smokers, have more comorbidities, co-medications, polypharmacy, and a greater number of primary care consultations (CPRD Aurum only) than DOAC users. However, they were less likely to be current alcohol users, and had recent prescriptions for beta-blockers, non-steroidal anti-inflammatory drugs (NSAIDs) and proton-pump inhibitor (PPI) than DOAC users. Notably, warfarin users were less likely to be obese, and have hypertension, a recent prescription for calcium channel blockers in CDARS (but not in CPRD Aurum).

The distribution of PSs is shown in Additional file 2, Table S1. After PS weighting, good covariate balances were achieved, with all standardised mean differences less than 0.05 in both settings (Additional file 3, Table S2).

**Fig. 2:**
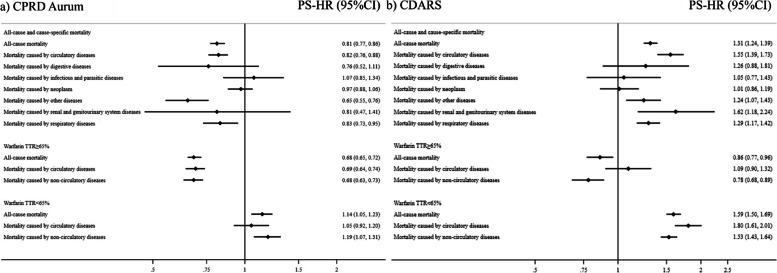
Pooled propensity score weighted estimates in the CPRD Aurum (**a**) and CDARS (**b**). Abbreviations: CPRD, Clinical Practice Research Datalink; CDARS, Clinical Data Analysis and Reporting System; PS-HR, propensity weighted hazard ratio; CI, confidence interval; TTR, time in therapeutic range. Reference group: DOACs

In CPRD Aurum, we found a decreased hazard of all-cause mortality in warfarin users (crude rate 69.64 per 1000 person-years) with a PS-weighted HR of 0.81 (95% CI 0.77–0.86), compared with DOAC users (crude rate 105.98 per 1000 person-years). In contrast, an increased hazard of all-cause mortality was associated with warfarin (crude rate 77.59 per 1000 person-years) with a PS-weighted HR of 1.31 (95% CI 1.24–1.39), compared with DOACs (crude rate 63.19 per 1000 person-years) in CDARS (Fig. 2 and Additional file 4, Tables S3–S4).

For the duration of effect, the decreased hazard of all-cause mortality associated with warfarin, compared with DOACs was observed in any follow-up periods in the English cohort which was consistent with the main finding. Likewise, results for each follow-up period were similar to the main analysis in the Hong Kong cohort. It is noted that although median follow-up in warfarin was twice as long as DOACs’ in both settings, similar findings were observed in either shorter follow-up or longer follow-up (Additional file 4, Tables S3–S4).

### Cause-specific mortality

In CPRD Aurum, warfarin users had a decreased hazard of mortality caused by circulatory diseases (PS-weighted HR: 0.82, 95% CI: 0.76–0.88), respiratory disease (PS-weighted HR: 0.83, 95% CI: 0.73–0.95) and other diseases compared with DOAC users (PS-weighted HR: 0.72, 95% CI: 0.63–0.82), but not for any specific causes of death (including digestive diseases, renal and genitourinary system disease, infectious and parasitic diseases, and neoplasms).

In CDARS, we found increased hazard of death due to renal and genitourinary system diseases (PS-weighted HR: 1.62, 95% CI: 1.18–2.24), circulatory diseases (PS-weighted HR: 1.55, 95% CI: 1.39–1.73), respiratory diseases (PS-weighted HR: 1.29, 95% CI: 1.17–1.42), and other diseases (PS-weighted HR: 1.24, 95% CI: 1.07–1.43) (Additional file 5, Tables S5–S6).

### Subgroup, secondary and sensitivity analyses

In line with the main analysis in both settings, similar patterns were found in most subgroups, which were lower hazard (CPRD Aurum), but higher hazard of all-cause mortality (CDARS) associated with warfarin, compared with DOACs (Additional file 6, Tables S7–S8).

When we stratified warfarin users by TTR, similar results were observed in both CPRD Aurum and CDARS. Warfarin users with TTR ≥ 65% had a lower hazard of all-cause mortality, compared with DOAC users (PS-weighted HR: 0.68, 95% CI: 0.65–0.72 in CPRD Aurum; PS-weighted HR: 0.86, 95% CI: 0.77–0.96 in CDARS), while an increased hazard of all-cause mortality was shown in warfarin users with TTR < 65%, compared with DOACs users (PS-weighted HR: 1.14, 95% CI: 1.05–1.23 in CPRD Aurum; PS-weighted HR: 1.59, 95% C: 1.50–1.69 in CDARS) (Fig. 2 and Additional file 7, Tables S9–S10). 15.32% and 0.44% warfarin users had missing INR records in CPRD Aurum and CDARS, respectively.

Upon further investigation into the potential observation of similar findings in circulatory and non-circulatory death under TTR stratification, comparing warfarin users with TTR ≥ 65% with DOAC users, we found lower hazards of both circulatory and non-circulatory death in CPRD Aurum; while in CDARS, there was no difference in the hazard of circulatory death, but a lower hazard of non-circulatory death was identified (Fig. 2 and Additional file 7, Tables S11–S14). We also observed an increased hazard of both circulatory and non-circulatory death associated with warfarin users with TTR < 65%, compared with DOAC users in CDARS, but only for non-circulatory death in CPRD Aurum.

In CPRD Aurum, compared with warfarin, increased hazards of all-cause mortality were found in rivaroxaban and dabigatran, while decreased hazard was observed in edoxaban. No evidence supported an association with apixaban. However, in CDARS, approximately a range of 18–43% lower hazard was found in different types of DOACs compared with warfarin (Additional file 7, Tables S15–S16).

All results of sensitivity analyses were similar to the main analysis in both CPRD and CDARS (Additional file 8, Table S17).

### Quantitative bias analyses

To potentially fully explain the PS-weighted HR (0.81) or the upper bound of the 95% CI (0.86) in CPRD Aurum and PS-weighted HR (1.31) or the lower bound of the 95% CI (1.24) in CDARS, an unmeasured confounder would need to be associated (conditional on measured covariates) with either DOACs, relative to warfarin use or mortality with a risk ratio of at least 1.77 (effect estimate) or 1.60 (lower bound) in CPRD Aurum and 1.95 (effect estimate) or 1.79 (lower bound) in CDARS (Additional file 9, Figs. S1–S2).

## Discussion

Using population-based electronic health records from England and Hong Kong, we observed a lower hazard of all-cause mortality associated with warfarin in CPRD Aurum, but an increased hazard in CDARS compared with DOACs. However, importantly, we consistently showed that the hazard of all-cause mortality associated with warfarin is largely dependent on anticoagulation control, measured by TTR in both databases in our exploratory analysis. Warfarin use with better controlled INR (TTR ≥ 65%) was associated with a lower hazard of all-cause mortality, compared with DOAC use; while warfarin use with poor INR control (TTR < 65%) was associated with an increased hazard of death, compared with DOAC use. Notably, this study is unable to establish a causal relationship between warfarin and mortality stratified by TTR, versus DOACs, due to the use of post-baseline measurements of INR.

To explore the role of INR control on all-cause mortality, we used both circulatory and non-circulatory mortality as outcomes in the stratified analysis by TTR. If the lower hazard of all-cause mortality associated with better INR-controlled warfarin users versus DOACs was mediated by pharmacological INR control alone, we would have observed similar patterns for circulatory mortality only but not non-circulatory mortality. However, we also observed a lower hazard of non-circulatory death comparing better INR-controlled warfarin users, with DOAC users. In addition, we observed a higher hazard of non-circulatory death comparing poor INR-controlled warfarin users with DOAC users in both settings, indicating that our results might be explained by unmeasured confounding. Notably, poor INR-controlled warfarin users versus DOAC users were more likely to be frailer (older, more comorbidities, co-medications and polypharmacy), leading to an increased hazard of non-circulatory death. Our findings therefore suggested that the difference in hazard of all-cause mortality between warfarin and DOACs reported in observational studies may be at least, in part, explained by unmeasured confounding, for example, due to disease severity. Similar findings were reported in a cohort study using data from a multi-national registry [38]. It showed that warfarin users with TTR < 65% had a 2.39-fold increased hazard (95% CI: 1.87–3.06) of all-cause mortality, compared with warfarin users with TTR ≥ 65% [38]. However, they had a small sample size (*n* = 9934), short follow-up (1 year) and did not investigate specific causes of mortality to investigate whether the associations were specific to circulatory mortality. In our study, TTR was calculated during follow-up, and we present these results for descriptive purposes only. To investigate the causal role of TTR on the risk of mortality would likely necessitate leveraging methods that appropriately handle time-dependent measurements [39].

It is noted that risks of specific causes of death associated with warfarin compared with DOACs were not consistently observed in CDARS and CPRD Aurum. In particular, a 62% increased hazard of death due to renal and genitourinary system disease was found comparing warfarin with DOACs in CDARS (95% CI: 1.18–2.24). It is possible that clinicians opt for warfarin in patients with renal diseases in Hong Kong, leading to possible channelling bias. In contrast, a previous study showed that warfarin-related nephropathy may accelerate the progression and increase the hazard of death [40]. Similar to the main analyses, decreased hazards of specific causes of death were observed in CPRD Aurum (respiratory [17%] and circulatory [18%]), although warfarin was usually reported with increased risk of mortality in people with pulmonary foundation diseases (e.g. pulmonary fibrosis) [41, 42] and it was reported that warfarin may increase the risk of bleeding, leading to circulatory death [43, 44]. Therefore, future studies are recommended to understand the role of warfarin compared with DOACs in cause-specific mortality, such as renal and genitourinary system disease death among people with renal diseases, respiratory disease death in people with pulmonary foundation diseases, or circulatory death in people with cardiovascular diseases.

Regarding individual DOAC, previous studies in the UK and Denmark concluded that individual DOAC (e.g. rivaroxaban and apixaban) was associated with a higher hazard of all-cause mortality in people with atrial fibrillation, compared with warfarin [12, 13]. This is similar to our primary finding but not in CDARS. Our previous work using another primary care database (i.e. OpenSAFELY) also reported a lower hazard of all-cause mortality associated with warfarin versus DOACs [45]. However, all studies had shorter follow-ups (< 6 years, 2.5 years and 7 months, respectively) and did not investigate the effects of potential residual confounding using stratified analysis by TTR.

Although the findings of this study do not support a causal association between all-cause mortality and warfarin, compared with DOACs, we identified high mortality rates in warfarin users with poor INR control. We therefore recommend that this patient group at risk, particularly Chinese patients, should be targeted for managing their medical condition (e.g. regular INR monitoring) to minimise the potential hazard of mortality, such as renal and genitourinary system death. While the lower mortality among DOAC users compared with warfarin users with poor control is likely to be caused in part by differences between the two patient groups, patients with persistently poor control on warfarin may possibly benefit from switching to a DOAC.

This is the first study using both a territory-wide Chinese EHR database and an English primary care database linked with hospital data, providing information on more than 191,000 people with good quality records to identify exposure, outcome and covariates to minimise confounding. Furthermore, our results can be generalised to both Caucasians and Chinese using both settings. We also stratified warfarin users by TTR, other potential subgroups, and investigated the duration of effect and cause-specific mortality to further understand the association between mortality and OACs. Although we could likely not eliminate residual confounding, we controlled for a wide range of potential confounders such as demographics, lifestyle factors, chronic comorbidities, and concomitant drugs in our analysis. We also included cause-specific mortality as outcomes to strengthen our interpretation of the results. Future studies could explore the use of high-dimensional propensity scores (HDPS) to control for confounding. However, if the additional variables included are not proxies for important unmeasured confounders, the HDPS might still not fully mitigate confounding [46, 47].

Nonetheless, there are some limitations in this study. Firstly, prescriptions issued in hospitals were not available in English data which may lead to an underestimation of exposure to OACs in the CPRD Aurum. However, primary care physicians maintain or continue prescriptions initially started in hospitals in England, which would be captured in CPRD Aurum. Further, CDARS contains hospital prescribing data which could complement the findings with CPRD Aurum. While lifestyle data such as BMI, smoking status and alcohol consumption were not recorded in CDARS, CPRD Aurum contains information on lifestyle factors to better control for confounders. Additionally, we did not have the data on whether the included individuals actually took the drugs as directed which may underestimate the association due to non-differential misclassification bias of exposure [48], similar to other classic observational studies using EHR databases. However, patients who were prescribed OACs usually have regular follow-ups and thus would have close monitoring in our databases. In addition, we used TTR as the proxy for drug adherence in warfarin users to support the interpretation of the study. Moreover, given that meaningful INR could only be obtained 3–4 days after starting each warfarin treatment [35], the analysis of TTR could be limited due to the post-baseline measure available for only one treatment arm leading to a risk of selection bias. Patients with TTR available may be healthier than those without this measure, given that patients have to survive and not be hospitalised to have INR measurements. Given the risk of selection bias in the analysis by TTR, these results should be considered exploratory and interpreted with caution.

## Conclusions

Our study does not support a causal association between all-cause mortality and warfarin users, compared with DOACs. Given the relatively high rates of mortality in warfarin users with poor INR control and the higher prevalence of poor INR control in Hong Kong, we recommend particularly Chinese patients in Hong Kong, should be targeted for managing their medical condition such as regularly monitoring INR, to reduce the risk of death.

## Supplementary Information

Additional file 1: Sects. 1.1–1.3 Section 1.1 Databases; Sect. 1.2 Covariates; Sect. 1.3 Time in Therapeutic Range calculations.

Additional file 2: Table. S1. Tables S1 Distribution of propensity scores.

Additional file 3: Table. S2. Table S2. Standardised mean difference before and after propensity score weighting in CPRD Aurum and CDARS.

Additional file 4: Table. S3-4. Table S3. Number of events, accumulated person-time, and unadjusted and propensity score weighted hazard ratios of all-cause mortality in warfarin and DOACs groups, CPRD Aurum – Main analysis and duration of effect. Table S4 Number of events, accumulated person-time, and unadjusted and propensity score weighted hazard ratios of all-cause mortality in warfarin and DOACs groups, CDARS – Main analysis and duration of effect.

Additional file 5: Table. S5-6. Table S5. Number of events, accumulated person-time, and unadjusted and propensity score weighted hazard ratios of cause-specific mortality in warfarin and DOACs groups, CPRD Aurum. Table S6 Number of events, accumulated person-time, and unadjusted and propensity score weighted hazard ratios of cause-specific mortality in warfarin and DOACs groups, CDARS.

Additional file 6: Table. S7-8. Table S7. Number of events, accumulated person-time, and unadjusted and propensity score weighted hazard ratios of all-cause mortality in warfarin and DOACs groups, CPRD Aurum—Subgroup analyses. Table S8. Number of events, accumulated person-time, and unadjusted and propensity score weighted hazard ratios of all-cause mortality in warfarin and DOACs groups, CDARS—Subgroup analyses.

Additional file 7: Table S9-16. Table S9. Number of events, accumulated person-time, and unadjusted and propensity score weighted hazard ratios of all-cause mortality in warfarin and DOACs groups, CPRD Aurum—Secondary analysis: warfarin TTR ≥ 65%, < 65% vs DOACs. Table S10. Number of events, accumulated person-time, and unadjusted and propensity score weighted hazard ratios of all-cause mortality in warfarin and DOACs groups, CDARS—Secondary analysis: warfarin TTR ≥ 65%, < 65% vs DOACs. Table S11. Number of events, accumulated person-time, and unadjusted and propensity score weighted hazard ratios of circulatory death in warfarin and DOACs groups, CPRD Aurum—Secondary post hoc analysis: warfarin TTR ≥ 65%, < 65% vs DOACs. Table S12. Number of events, accumulated person-time, and unadjusted and propensity score weighted hazard ratios of non-circulatory death in warfarin and DOACs groups, CPRD Aurum—Secondary post hoc analysis: warfarin TTR ≥ 65%, < 65% vs DOACs. Table S13. Number of events, accumulated person-time, and unadjusted and propensity score weighted hazard ratios of circulatory death in warfarin and DOACs groups, CDARS—Secondary post hoc analysis: warfarin TTR ≥ 65%, < 65% vs DOACs. Table S14. Number of events, accumulated person-time, and unadjusted and propensity score weighted hazard ratios of non-circulatory death in warfarin and DOACs groups, CDARS—Secondary post hoc analysis: warfarin TTR ≥ 65%, < 65% vs DOACs. Table S15 Number of events, accumulated person-time, and unadjusted and propensity score weighted hazard ratios of all-cause mortality in warfarin and DOACs groups, CPRD Aurum—Secondary analysis: warfarin vs individual DOACs. Table S16 Number of events, accumulated person-time, and unadjusted and propensity score weighted hazard ratios of all-cause mortality in warfarin and DOACs groups, CDARS—Secondary analysis: warfarin vs individual DOACs.

Additional file 8: Table S17. Table S17. Sensitivity analyses.

Additional file 9: Figure S1-2.Figure S1. E-value for the lower 95% confidence interval and point estimate in atrial fibrillation with anticoagulant use in CPRD Aurum. Figure S2. E-value for the lower 95% confidence interval and point estimate in atrial fibrillation with anticoagulant use in CDARS.

## Data Availability

No additional data available.

## Abbreviations

OACs: Oral anticoagulants
DOACs: Direct oral anticoagulants
UK: United Kingdom
INR: International normalised ratio
TTR: Time in therapeutic range
GPs: General practitioners
CPRD: Clinical Practice Research Datalink
ONS: Office for National Statistics
HES APC: Hospital Episode Statistics Admitted Patient Care
IMD: Index of Multiple Deprivation
CDARS: Clinical Data Analysis and Reporting System
HA: Hospital Authority
EHRs: Electronic health records
eGFR: Estimated glomerular filtration rate
ICD-10: International Classification of Diseases, Tenth Revision
HR: Hazard ratio
CI: Confidence interval
PS: Propensity score
IPTW: Inverse probability of treatment weights
BMI: Body mass index
IQR: Interquartile range
NSAIDs: Non-steroidal anti-inflammatory drugs
PPI: Proton-pump inhibitor
N: Number
