# Supplementary material for: Risk of mortality between warfarin and direct oral anticoagulants: population-based cohort studies
Source: BMC Med. 2024 Dec 23;22:597. doi: 10.1186/s12916-024-03808-y (PMC11664815; doi:10.1186/s12916-024-03808-y)
Supplement: Supplementary file 1 — Additional file 1: Sects. 1.1–1.3 Section 1.1 Databases; Sect. 1.2 Covariates; Sect. 1.3 Time in Therapeutic Range calculations. [file 12916_2024_3808_MOESM1_ESM.docx]

**Additional file 1 Details of databases, covariates, and Time in Therapeutic Range calculations**

1.1 Databases:

England: The Clinical Practice Research Datalink (CPRD) contains anonymised primary care electronic health records (EHRs) from general practitioners (GPs). Information on demographics, lifestyle behaviours (smoking and alcohol consumption) and anthropometric data (height and weight), diagnoses (coded using Systematised Nomenclature of Medicine Clinical Terms [SNOMED], Read and local Egton Medical Information Systems [EMIS] codes), prescriptions (coded using British National Formulary [BNF] codes), laboratory test results and referrals to specialists are recorded in CPRD. We used CPRD Aurum (May 2022 Build) which contains 41,200,722 acceptable patients with approximately 20% United Kingdom population coverage. Diagnoses and procedures recorded in HES were recorded from National Health Service (NHS) England, coded using the International Classification of Diseases, Tenth Revision (ICD-10) and UK Office of Population, Census and Surveys classification (OPCS).

Hong Kong: Demographics, diagnosis (coded using ICD, Ninth Revision [ICD-9]), medication prescribing and dispensing records (coded according to BNF), laboratory results, and cause of death (coded using ICD-10) are recorded in Clinical Data Analysis and Reporting System (CDARS).

1.2 Covariates:

The following variables were considered as covariates for controlling confounding in both propensity score calculation and multivariable regression: age at index date, gender, ethnicity (CPRD Aurum only; 92% were Chinese in CDARS), calendar year of index date, body mass index (BMI [underweight <18.5kg/m^2^, normal weight 18.5-24.9kg/m^2^, overweight 25-29.9kg/m^2^, obese ≥30kg/m^2^] in CPRD Aurum; diagnosis of overweight, obesity or other related lipid metabolism disorders as proxy in CDARS), smoking status (current/ex-/non-smoker in CPRD Aurum, chronic obstructive pulmonary disease as proxy in CDARS), alcohol consumption (current/ex-/non-drinker in CPRD Aurum, alcohol related disorders as proxy in CDARS), hypertension (calculated using blood pressure in CPRD Aurum; diagnosis of hypertensive disorders as proxy in CDARS), CHA2DS2-VASc score, HAS-BLED score (we used diagnoses for components identification and calculate them using formulas. It is noted that we use modified HAS-BLED as proxy, excluding labile INR element from the HAS-BLED score, as DOACs group does not contain INR measurements), diabetes mellitus, heart failure, peripheral artery disease, ischaemic heart disease, venous thromboembolism, ischemic stroke/transient ischemic attack, chronic renal failure (in addition to relevant diagnostic codes, identified by renal function serum creatinine laboratory test corresponding to an eGFR <60mL/min/1.73m^2^ or ≥60mL/min/1.73m^2^, eGFR calculated using the Chronic Kidney Disease Epidemiology Collaboration equation. For those who have normal kidney function, their serum creatinine levels were unlikely to be measured and recorded in the routine clinical setting. Therefore, we assumed that people without serum creatinine measurement were considered to have normal kidney function]), liver disease, gastrointestinal bleeding, intracranial bleeding, other bleeding, co-medications used within 90 days prior to index date (Angiotensin-Converting Enzyme Inhibitors [ACEI]/Angiotensin Receptor Blocker [ARB], antiarrhythmics, antiplatelets, aspirin, beta-blocker, calcium-channel blocker, Histamine Type-2 Receptor Antagonists/Blockers [H2 blocker], Non-Steroidal Anti-inflammatory Drugs [NSAIDS], or Proton-Pump Inhibitor [PPI]), polypharmacy , IMD (CPRD Aurum only). Due to the difference in healthcare systems, we used active contact in primary care in the past year in CPRD Aurum only as a measure of healthcare seeking behaviour. Diagnostic code list can be found through Data Compass <https://doi.org/10.17037/DATA.00003652>.

1.3 Time in Therapeutic Range calculations:

The Rosendaal method was used for TTR calculation, assuming that INR changes the same amount each day: 1) calculate quantity of the total shift within the therapeutic range (2.0-3.0), 2) calculate percentage of the total shift within the therapeutic range, and 3) estimate number of days within the therapeutic range since last visit. To calculate the overall percentage in range, sum the total days within range for each time period and divide by the total number of treatment days. TTR was not considered as a baseline covariate and was calculated using INR data during follow-up period.
