# Supplementary material for: Risk of mortality between warfarin and direct oral anticoagulants: population-based cohort studies
Source: BMC Med. 2024 Dec 23;22:597. doi: 10.1186/s12916-024-03808-y (PMC11664815; doi:10.1186/s12916-024-03808-y)
Supplement: Supplementary file 2 — Additional file 2: Table. S1. Tables S1 Distribution of propensity scores. [file 12916_2024_3808_MOESM2_ESM.docx]

**Additional file 2 Distribution of propensity scores**

**Table S1 Distribution of propensity scores**

|  | **CPRD Aurum** | | | | **CDARS** | | | |
| --- | --- | --- | --- | --- | --- | --- | --- | --- |
|  | **DOACs users (N=80,001)** | | **Warfarin users (N=72,855)** | | **DOACs users (N=25,179)** | | **Warfarin users (N=12,897)** | |
| **Propensity score in 20 groups*** | **N (%)** | **Death (%)** | **N (%)** | **Death (%)** | **N (%)** | **Death (%)** | **N (%)** | **Death (%)** |
| **1** | 7312 (9.14) | 1129 (5.75) | 331 (0.45) | 40 (0.16) | 1812 (7.20) | 73 (1.86) | 92 (0.71) | 8 (0.20) |
| **2** | 7183 (8.98) | 1153 (5.87) | 460 (0.63) | 47 (0.19) | 1719 (6.83) | 78 (1.98) | 185 (1.43) | 20 (0.49) |
| **3** | 7104 (8.88) | 974 (4.96) | 539 (0.74) | 47 (0.19) | 1708 (6.78) | 133 (3.38) | 196 (1.52) | 27 (0.66) |
| **4** | 6989 (8.74) | 853 (4.34) | 655 (0.90) | 66 (0.26) | 1640 (6.51) | 143 (3.64) | 264 (2.05) | 36 (0.88) |
| **5** | 6764 (8.45) | 1495 (7.61) | 877 (1.20) | 169 (0.68) | 1642 (6.52) | 184 (4.68) | 261 (2.02) | 49 (1.20) |
| **6** | 6543 (8.18) | 1558 (7.93) | 1100 (1.51) | 212 (0.85) | 1600 (6.35) | 189 (4.81) | 304 (2.36) | 46 (1.13) |
| **7** | 6161 (7.70) | 1466 (7.46) | 1482 (2.03) | 280 (1.12) | 1579 (6.27) | 225 (5.72) | 325 (2.52) | 60 (1.47) |
| **8** | 5715 (7.14) | 1950 (9.93) | 1928 (2.65) | 562 (2.25) | 1488 (5.91) | 182 (4.63) | 416 (3.23) | 92 (2.26) |
| **9** | 5323 (6.65) | 1297 (6.60) | 2320 (3.18) | 442 (1.77) | 1502 (5.97) | 222 (5.64) | 402 (3.12) | 68 (1.67) |
| **10** | 4642 (5.80) | 1828 (9.30) | 3000 (4.12) | 1036 (4.15) | 1447 (5.75) | 240 (6.10) | 456 (3.54) | 98 (2.41) |
| **11** | 3927 (4.91) | 1365 (6.95) | 3716 (5.10) | 1059 (4.25) | 1371 (5.45) | 243 (6.18) | 533 (4.13) | 138 (3.39) |
| **12** | 3461 (4.33) | 1007 (5.13) | 4182 (5.74) | 1021 (4.09) | 1325 (5.26) | 253 (6.43) | 579 (4.49) | 142 (3.49) |
| **13** | 2574 (3.22) | 1250 (6.36) | 5069 (6.96) | 2106 (8.45) | 1197 (4.75) | 296 (7.53) | 707 (5.48) | 200 (4.91) |
| **14** | 2117 (2.65) | 695 (3.54) | 5526 (7.58) | 1505 (6.04) | 1115 (4.43) | 267 (6.79) | 789 (6.12) | 202 (4.96) |
| **15** | 1650 (2.06) | 678 (3.45) | 5992 (8.22) | 2273 (9.11) | 1065 (4.23) | 273 (6.94) | 838 (6.50) | 270 (6.63) |
| **16** | 1134 (1.42) | 369 (1.88) | 6509 (8.93) | 2105 (8.44) | 959 (3.81) | 279 (7.09) | 945 (7.33) | 319 (7.83) |
| **17** | 811 (1.01) | 362 (1.84) | 6832 (9.38) | 2902 (11.64) | 823 (3.27) | 242 (6.15) | 1081 (8.38) | 369 (9.06) |
| **18** | 428 (0.53) | 169 (0.86) | 7215 (9.90) | 2624 (10.52) | 595 (2.36) | 186 (4.73) | 1309 (10.15) | 514 (12.62) |
| **19** | 129 (0.16) | 36 (0.18) | 7514 (10.31) | 3693(14.81) | 381 (1.51) | 147 (3.74) | 1523 (11.81) | 624 (15.32) |
| **20** | 34 (0.04) | 12 (0.06) | 7608 (10.44) | 2748 (11.02) | 211 (0.84) | 78 (1.98) | 1692 (13.12) | 790 (19.40) |

*1 = lowest likelihood of receiving oral anticoagulant; 2 = highest likelihood of receiving oral anticoagulant; based on factors included in propensity score

Abbreviations: CPRD = Clinical Research Practice Datalink, CDARS = Clinical Data Analysis and Reporting System, DOAC = direct oral anticoagulant
