# Supplementary material for: Risk of mortality between warfarin and direct oral anticoagulants: population-based cohort studies
Source: BMC Med. 2024 Dec 23;22:597. doi: 10.1186/s12916-024-03808-y (PMC11664815; doi:10.1186/s12916-024-03808-y)
Supplement: Supplementary file 3 — Additional file 3: Table. S2. Table S2. Standardised mean difference before and after propensity score weighting in CPRD Aurum and CDARS. [file 12916_2024_3808_MOESM3_ESM.docx]

**Additional file 3 Standardised mean difference before and after propensity score weighting**

**Table S2 Standardised mean difference before and after propensity score weighting in CPRD Aurum and CDARS**

| **Covariates** | **CPRD Aurum** | | **CDARS** | |
| --- | --- | --- | --- | --- |
|  | Before PS weighting SMD | After PS weighting SMD | Before PS weighting SMD | After PS weighting SMD |
| **Age group** |  |  |  |  |
| 18-<40 | 0.0180 | 0.0114 | 0.0924 | -0.0040 |
| 40-<50 | 0.0374 | -0.0145 | 0.1232 | -0.0012 |
| 50-<60 | 0.0327 | 0.0283 | 0.1751 | 0.0129 |
| 60-<70 | 0.0917 | 0.0053 | 0.0800 | 0.0088 |
| 70-<80 | 0.0744 | -0.0349 | -0.0169 | -0.0062 |
| 80+ | -0.1763 | 0.0192 | -0.2155 | -0.0079 |
| **Gender (Female)** | -0.0178 | 0.0081 | -0.0979 | -0.0071 |
| **Calendar year at cohort entry** |  |  |  |  |
| 2011 | 0.6435 | -0.0190 | 0.6197 | -0.0225 |
| 2012 | 0.6322 | 0.0057 | 0.2939 | -0.0030 |
| 2013 | 0.5013 | 0.0019 | 0.2205 | 0.0082 |
| 2014 | 0.2924 | 0.0008 | 0.1667 | 0.0057 |
| 2015 | -0.0412 | 0.0006 | 0.0459 | 0.0026 |
| 2016 | -0.3395 | 0.0029 | -0.0754 | 0.0043 |
| 2017 | -0.5114 | -0.0031 | -0.2070 | 0.0061 |
| 2018 | -0.5912 | -0.0007 | -0.3008 | 0.0033 |
| 2019 | -0.5726 | 0.0093 | -0.5381 | -0.0053 |
| **CHA2DS2 VASc score** | -0.1735 | 0.0124 | -0.0470 | -0.0250 |
| **HASBLED score** | -0.1168 | -0.0118 | -0.0603 | -0.0207 |
| **BMI category** |  |  |  |  |
| Underweight | -0.0624 | 0.0030 | NA | NA |
| Normal weight | -0.0643 | 0.0189 | NA | NA |
| Overweight | 0.0241 | -0.0285 | NA | NA |
| Obese | 0.0532 | 0.0102 | NA | NA |
| **Overweight/obesity/other related lipid metabolism disorders** | NA | NA | -0.0376 | -0.0141 |
| **Smoking status** |  |  |  |  |
| Non-smoker | 0.0309 | -0.0003 | NA | NA |
| Current smoker | 0.0287 | -0.0306 | NA | NA |
| Ex-smoker | -0.0497 | 0.0260 | NA | NA |
| **Chronic obstructive pulmonary disease** | NA | NA | -0.0051 | 0.0026 |
| **Alcohol consumption status** |  |  |  |  |
| Non-alcohol user | -0.0054 | 0.0075 | NA | NA |
| Current alcohol user | -0.0336 | -0.0158 | NA | NA |
| Ex-alcohol user | 0.0447 | 0.0130 | NA | NA |
| **Alcohol related disorders** | NA | NA | 0.0343 | 0.0072 |
| **Ethnicity** |  |  |  |  |
| White | 0.0029 | 0.0048 | NA | NA |
| South Asian | 0.0005 | 0.0094 | NA | NA |
| Black | -0.0019 | -0.0139 | NA | NA |
| Other | 0.0032 | 0.0018 | NA | NA |
| Mixed | -0.0016 | 0.0069 | NA | NA |
| Not stated | -0.0142 | 0.0002 | NA | NA |
| **Index of Multiple Deprivation** |  |  |  |  |
| Quintile 1 (least deprived) | -0.0028 | -0.0038 | NA | NA |
| Quintile 2 | -0.0003 | -0.0137 | NA | NA |
| Quintile 3 | 0.0069 | -0.0042 | NA | NA |
| Quintile 4 | -0.0013 | 0.0037 | NA | NA |
| Quintile 5 (most deprived) | -0.0025 | 0.0209 | NA | NA |
| **Category of GP consultation within 1 year before cohort entry** |  |  |  |  |
| 12+ visits | 0.0438 | -0.0069 | NA | NA |
| 1-11 visit(s) | -0.0664 | 0.0093 | NA | NA |
| 0 visit | 0.0676 | -0.0065 | NA | NA |
| **Polypharmacy (≥5 drugs) within 90 days before cohort entry** |  |  |  |  |
| 0 | NA | NA | -0.4676 | -0.0001 |
| 1-4 | 0.0033 | -0.0038 | 0.1549 | 0.0252 |
| 5-9 | 0.0415 | 0.0060 | 0.1414 | 0.0030 |
| ≥10 | -0.0468 | -0.0033 | 0.1896 | -0.0207 |
| **Comorbidities at cohort entry** |  |  |  |  |
| Bleeding - gastrointestinal bleeding | -0.1247 | 0.0064 | 0.0174 | -0.0035 |
| Bleeding - intracranial haemorrhage | -0.0494 | 0.0109 | 0.0177 | 0.0016 |
| Bleeding - other bleeding | -0.1633 | -0.0117 | -0.0007 | 0.0012 |
| Chronic renal failure | -0.0154 | 0.0023 | 0.2816 | -0.0416 |
| Diabetes mellitus | -0.0715 | 0.0013 | 0.0134 | -0.0013 |
| Heart failure | -0.0580 | 0.0284 | 0.2247 | -0.0205 |
| Hypertension* | -0.0459 | -0.0009 | -0.0269 | -0.0153 |
| Ischaemic heart disease | -0.0371 | 0.0014 | 0.0699 | -0.0256 |
| Ischemic stroke or transient ischemic attacks | -0.1279 | 0.0048 | -0.0012 | -0.0100 |
| Liver disease | -0.0864 | 0.0076 | 0.0525 | -0.0043 |
| Peripheral artery disease | -0.0219 | 0.0071 | 0.0594 | 0.0020 |
| Venous thromboembolism | -0.0112 | 0.0230 | 0.2251 | 0.0101 |
| **Co** **-medications within 90 days before cohort entry** |  |  |  |  |
| ACEI/ARB | 0.0681 | -0.0116 | 0.0064 | -0.0059 |
| Antiarrhythmics | 0.0701 | -0.0016 | 0.0204 | 0.0007 |
| Antiplatelets | -0.0891 | -0.0195 | 0.0055 | -0.0177 |
| Aspirin | 0.2869 | 0.0027 | -0.0010 | -0.0158 |
| Beta-blockers | -0.0213 | -0.0001 | -0.0585 | -0.0019 |
| Calcium channel blockers | 0.0207 | -0.0122 | -0.1181 | 0.0011 |
| H2 blockers | -0.0941 | 0.0098 | 0.0047 | -0.0015 |
| NSAIDS | -0.0188 | -0.0124 | -0.0104 | 0.0018 |
| PPI | -0.0766 | -0.0078 | -0.1102 | -0.0119 |

Abbreviations**:** SMD = standardised mean difference, CPRD = Clinical Research Practice Datalink, CDARS = Clinical Data Analysis and Reporting System, PS = propensity score, BMI = body mass index, GP = general practice, ACEI = Angiotensin-Converting Enzyme Inhibitors; ARB = Angiotensin Receptor Blocker; H2 blocker = Histamine Type-2 Receptor Antagonists/Blockers, NSAIDs = Non-Steroidal Anti-inflammatory Drugs; PPI = Proton-Pump Inhibitor, NA = not applicable
