# Supplementary material for: Risk of mortality between warfarin and direct oral anticoagulants: population-based cohort studies
Source: BMC Med. 2024 Dec 23;22:597. doi: 10.1186/s12916-024-03808-y (PMC11664815; doi:10.1186/s12916-024-03808-y)
Supplement: Supplementary file 4 — Additional file 4: Table. S3-4. Table S3. Number of events, accumulated person-time, and unadjusted and propensity score weighted hazard ratios of all-cause mortality in warfarin and DOACs groups, CPRD Aurum – Main analysis and duration of effect. Table S4 Number of events, accumulated person-time, and unadjusted and propensity score weighted hazard ratios of all-cause mortality in warfarin and DOACs groups, CDARS – Main analysis and duration of effect. [file 12916_2024_3808_MOESM4_ESM.docx]

**Additional file 4 Tables of main analysis and duration of effect**

**Table S3 Number of events, accumulated person-time, and unadjusted and propensity score weighted hazard ratios of all-cause mortality in warfarin and DOACs groups, CPRD Aurum – Main analysis and duration of effect**

| **Follow-up period** | **Warfarin users** | | | | **DOACs users** | | | | **Unadjusted HR (95% CI)** | **Propensity score weighted HR (95% CI)** |
| --- | --- | --- | --- | --- | --- | --- | --- | --- | --- | --- |
|  | **Number of persons** | **Number of events** | **Person-years at risk** | **Rate per 1,000** | **Number of persons** | **Number of events** | **Person-years at risk** | **Rate per 1,000** |  |  |
| **0-≥5** | 73,178 | 25,021 | 359,294.09 | 69.64 | 80,057 | 19,658 | 185,494.62 | 105.98 | **0.64 (0.63, 0.65)** | **0.81 (0.77, 0.86)** |
| **0-<1** | 73,178 | 4,683 | 72,454.69 | 64.63 | 80,057 | 7,961 | 75,227.41 | 105.83 | **0.62 (0.60, 0.65)** | **0.78 (0.73, 0.84)** |
| **0-<2** | 73,178 | 8,739 | 137,900.43 | 63.37 | 80,057 | 12,732 | 141,523.13 | 89.96 | **0.71 (0.69, 0.73)** | **0.77 (0.73, 0.82)** |
| **0-<3** | 73,178 | 12,613 | 199,230.58 | 63.31 | 80,057 | 15,990 | 202,048.24 | 79.14 | **0.81 (0.79, 0.83)** | **0.80 (0.76, 0.85)** |
| **0-<4** | 73,178 | 16,371 | 256,600.46 | 63.80 | 80,057 | 18,048 | 258,414.73 | 69.84 | **0.92 (0.90, 0.94)** | **0.80 (0.76, 0.84)** |
| **0-<5** | 73,178 | 19,584 | 310,091.02 | 63.16 | 80,057 | 19,100 | 311,829.36 | 61.25 | **1.04 (1.02, 1.06)** | **0.82 (0.78, 0.86)** |

Reference group: DOAC users

Abbreviations: CPRD = Clinical Research Practice Datalink, DOAC = direct oral anticoagulant, HR = hazard ratio, CI = confidence interval

**Table S4 Number of events, accumulated person-time, and unadjusted and propensity score weighted hazard ratios of all-cause mortality in warfarin and DOACs groups, CDARS – Main analysis and duration of effect**

| **Follow-up period** | **Warfarin users** | | | | **DOACs users** | | | | **Unadjusted HR (95% CI)** | **Propensity score weighted HR (95% CI)** |
| --- | --- | --- | --- | --- | --- | --- | --- | --- | --- | --- |
|  | **Number of persons** | **Number of events** | **Person-years at risk** | **Rate per 1,000** | **Number of persons** | **Number of events** | **Person-years at risk** | **Rate per 1,000** |  |  |
| **0-≥5** | 13,068 | 4,187 | 53,960.57 | 77.59 | 25,233 | 3,935 | 62,271.49 | 63.19 | **1.28 (1.22, 1.34)** | **1.31 (1.24, 1.39)** |
| **0-<1** | 13,068 | 1,338 | 13,240.44 | 101.05 | 25,233 | 1,528 | 24,335.69 | 62.79 | **1.73 (1.61, 1.86)** | **1.58 (1.44, 1.75)** |
| **0-<2** | 13,068 | 2,028 | 24,175.85 | 83.89 | 25,233 | 2,327 | 47,025.01 | 49.48 | **1.74 (1.64, 1.84)** | **1.53 (1.41, 1.65)** |
| **0-<3** | 13,068 | 2,595 | 34,415.27 | 75.40 | 25,233 | 2,933 | 68,668.81 | 42.71 | **1.78 (1.69, 1.87)** | **1.42 (1.33, 1.52)** |
| **0-<4** | 13,068 | 3,049 | 44,014.31 | 69.27 | 25,233 | 3,365 | 89,488.12 | 37.60 | **1.83 (1.75, 1.93)** | **1.37 (1.29, 1.46)** |
| **0-<5** | 13,068 | 3,422 | 53,126.67 | 64.29 | 25,233 | 3,624 | 109,711.07 | 33.03 | **1.92 (1.84, 2.02)** | **1.36 (1.28, 1.45)** |

Reference group: DOAC users

Abbreviations: CDARS = Clinical Data Analysis and Reporting System, DOAC = direct oral anticoagulant, HR = hazard ratio, CI = confidence interval
