# Supplementary material for: Risk of mortality between warfarin and direct oral anticoagulants: population-based cohort studies
Source: BMC Med. 2024 Dec 23;22:597. doi: 10.1186/s12916-024-03808-y (PMC11664815; doi:10.1186/s12916-024-03808-y)
Supplement: Supplementary file 5 — Additional file 5: Table. S5-6. Table S5. Number of events, accumulated person-time, and unadjusted and propensity score weighted hazard ratios of cause-specific mortality in warfarin and DOACs groups, CPRD Aurum. Table S6 Number of events, accumulated person-time, and unadjusted and propensity score weighted hazard ratios of cause-specific mortality in warfarin and DOACs groups, CDARS. [file 12916_2024_3808_MOESM5_ESM.docx]

**Additional file 5 Tables of cause-specific mortality risk**

**Table S5 Number of events, accumulated person-time, and unadjusted and propensity score weighted hazard ratios of cause-specific mortality in warfarin and DOACs groups, CPRD Aurum**

| **Cause-specific** | **Warfarin users** | | | | **DOACs users** | | | | **Unadjusted HR (95% CI)** | **Propensity score weighted HR (95% CI)** |
| --- | --- | --- | --- | --- | --- | --- | --- | --- | --- | --- |
|  | **Number of persons** | **Number of events** | **Person-years at risk** | **Rate per 1,000** | **Number of persons** | **Number of events** | **Person-years at risk** | **Rate per 1,000** |  |  |
| **Respiratory diseases** | 73,178 | 4,293 | 359,237.34 | 11.95 | 80,057 | 3,480 | 185,450.33 | 18.77 | **0.65 (0.62, 0.68)** | **0.83 (0.73, 0.95)** |
| **Circulatory diseases** | 73,178 | 9,460 | 359,251.49 | 26.33 | 80,057 | 7,421 | 185,461.12 | 40.01 | **0.66 (0.64, 0.68)** | **0.82 (0.76, 0.88)** |
| **Other diseases** | 73,178 | 4,125 | 359,236.88 | 11.48 | 80,057 | 3,556 | 185,450.54 | 19.17 | **0.49 (0.47, 0.52)** | **0.65 (0.55, 0.76)** |
| **Digestive diseases** | 73,178 | 1,023 | 359,228.39 | 2.85 | 80,057 | 814 | 185,443.03 | 4.39 | **0.65 (0.59, 0.72)** | 0.76 (0.52, 1.11) |
| **Renal and genitourinary system disease** | 73,178 | 552 | 359,227.10 | 1.54 | 80,057 | 403 | 185,441.90 | 2.17 | **0.69 (0.60, 0.79)** | 0.81 (0.47, 1.41) |
| **Neoplasms** | 73,178 | 5,283 | 359,240.05 | 14.71 | 80,057 | 3,766 | 185,451.11 | 20.31 | **0.72 (0.69, 0.75)** | 0.97 (0.88, 1.06) |
| **Infectious and parasitic diseases** | 73,178 | 285 | 359,226.37 | 0.80 | 80,057 | 218 | 185,441.10 | 1.18 | **0.69 (0.57, 0.83)** | 1.07 (0.85, 1.34) |

Reference group: DOAC users

Abbreviations: CPRD = Clinical Research Practice Datalink, DOAC = direct oral anticoagulant, HR = hazard ratio, CI = confidence interval

**Table S6 Number of events, accumulated person-time, and unadjusted and propensity score weighted hazard ratios of cause-specific mortality in warfarin and DOACs groups, CDARS**

| **Cause-specific** | **Warfarin users** | | | | **DOACs users** | | | | **Unadjusted HR (95% CI)** | **Propensity score weighted HR (95% CI)** |
| --- | --- | --- | --- | --- | --- | --- | --- | --- | --- | --- |
|  | **Number of persons** | **Number of events** | **Person-years at risk** | **Rate per 1,000** | **Number of persons** | **Number of events** | **Person-years at risk** | **Rate per 1,000** |  |  |
| **Renal and genitourinary system diseases** | 13,068 | 227 | 53,949.73 | 4.21 | 25,233 | 137 | 62,261.09 | 2.20 | **1.96 (1.58, 2.44)** | **1.62 (1.18, 2.24)** |
| **Circulatory diseases** | 13,068 | 1,288 | 53,952.64 | 23.87 | 25,233 | 994 | 62,263.44 | 15.96 | **1.61 (1.48, 1.75)** | **1.55 (1.39, 1.73)** |
| **Respiratory diseases** | 13,068 | 1,408 | 53,952.96 | 26.10 | 25,233 | 1,494 | 62,264.81 | 23.99 | **1.13 (1.04, 1.21)** | **1.29 (1.17, 1.42)** |
| **Other diseases** | 13,068 | 579 | 53,950.69 | 10.73 | 25,233 | 563 | 62,262.26 | 9.04 | **1.21 (1.07, 1.36)** | **1.24 (1.07, 1.43)** |
| **Digestive diseases** | 13,068 | 141 | 53,949.50 | 2.61 | 25,233 | 129 | 62,261.07 | 2.07 | 1.23 (0.96, 1.58) | 1.26 (0.88, 1.81) |
| **Infectious and parasitic diseases** | 13,068 | 122 | 53,949.44 | 2.26 | 25,233 | 135 | 62,261.09 | 2.17 | 1.11 (0.86, 1.42) | 1.05 (0.77, 1.43) |
| **Neoplasms** | 13,068 | 422 | 53,950.26 | 7.82 | 25,233 | 483 | 62,262.04 | 7.76 | 1.05 (0.92, 1.20) | 1.01 (0.86, 1.19) |

Reference group: DOAC users

Abbreviations: CDARS = Clinical Data Analysis and Reporting System, DOAC = direct oral anticoagulant, HR = hazard ratio, CI = confidence interval
