# Supplementary material for: Risk of mortality between warfarin and direct oral anticoagulants: population-based cohort studies
Source: BMC Med. 2024 Dec 23;22:597. doi: 10.1186/s12916-024-03808-y (PMC11664815; doi:10.1186/s12916-024-03808-y)
Supplement: Supplementary file 6 — Additional file 6: Table. S7-8. Table S7. Number of events, accumulated person-time, and unadjusted and propensity score weighted hazard ratios of all-cause mortality in warfarin and DOACs groups, CPRD Aurum—Subgroup analyses. Table S8. Number of events, accumulated person-time, and unadjusted and propensity score weighted hazard ratios of all-cause mortality in warfarin and DOACs groups, CDARS—Subgroup analyses. [file 12916_2024_3808_MOESM6_ESM.docx]

**Additional file 6 Tables of subgroup analyses**

**Table S7 Number of events, accumulated person-time, and unadjusted and propensity score weighted hazard ratios of all-cause mortality in warfarin and DOACs groups, CPRD Aurum - Subgroup analyses**

|  | **Warfarin users** | | | | **DOACs users** | | | | **Unadjusted HR (95% CI)** | **Propensity score weighted HR (95% CI)** |
| --- | --- | --- | --- | --- | --- | --- | --- | --- | --- | --- |
|  | **Number of persons** | **Number of events** | **Person-years at risk** | **Rate per 1,000** | **Number of persons** | **Number of events** | **Person-years at risk** | **Rate per 1,000** |  |  |
| ***Age*** |  |  |  |  |  |  |  |  |  |  |
| **18-<40** | 297 | 17 | 1,816.02 | 9.36 | 236 | 9 | 597.90 | 15.05 | 0.56 (0.25, 1.26) | **0.33 (0.13, 0.84)** |
| **40-<50** | 1,348 | 84 | 8,255.64 | 10.17 | 1,095 | 42 | 3,021.79 | 13.90 | **0.66 (0.46, 0.96)** | 1.58 (0.70, 3.54) |
| **50-<60** | 4,809 | 446 | 27,780.53 | 16.05 | 4.590 | 315 | 11,674.52 | 26.98 | **0.54 (0.47, 0.62)** | **0.68 (0.51, 0.89)** |
| **60-<70** | 15,288 | 2,421 | 86,153.86 | 28.10 | 13,797 | 1,437 | 35,934.96 | 39.99 | **0.64 (0.60, 0.69)** | **0.78 (0.64, 0.94)** |
| **70-<80** | 27,143 | 8,151 | 138,103.00 | 59.02 | 26,805 | 4,953 | 64,949.93 | 76.26 | **0.71 (0.69, 0.74)** | **0.78 (0.70, 0.88)** |
| **80+** | 24,293 | 13,902 | 97,185.04 | 143.05 | 33,534 | 12,902 | 69,315.52 | 186.13 | **0.72 (0.70, 0.74)** | **0.82 (0.77, 0.88)** |
| ***Sex*** |  |  |  |  |  |  |  |  |  |  |
| **Male** | 41,126 | 13,667 | 202,838.44 | 67.38 | 44,267 | 10,200 | 103,149.62 | 98.89 | **0.66 (0.65, 0.68)** | **0.88 (0.81, 0.95)** |
| **Female** | 32,052 | 11,354 | 156,455.65 | 72.57 | 35,790 | 9,458 | 82,345.00 | 114.86 | **0.61 (0.60, 0.63)** | **0.74 (0.69, 0.79)** |
| ***Calendar year of first OAC Rx date*** |  |  |  |  |  |  |  |  |  |  |
| **Before 2017** | 68,570 | 24,465 | 351,305.97 | 69.64 | 35,896 | 12,755 | 124,978.80 | 102.06 | **0.66 (0.65, 0.68)** | **0.83 (0.78, 0.89)** |
| **After 2017 (inclusive)** | 4,608 | 556 | 7,988.12 | 69.60 | 44,161 | 6,903 | 60,515.82 | 114.07 | **0.62 (0.57, 0.67)** | **0.72 (0.65, 0.79)** |
| ***Chronic kidney disease*** |  |  |  |  |  |  |  |  |  |  |
| **No** | 55,216 | 15,869 | 283,120.35 | 56.05 | 59,887 | 12,523 | 142,204.13 | 88.06 | **0.61 (0.60, 0.63)** | **0.79 (0.74, 0.85)** |
| **Yes** | 17,962 | 9,152 | 76,173.74 | 120.15 | 20,170 | 7,135 | 43,290.49 | 164.82 | **0.71 (0.68, 0.73)** | **0.87 (0.80, 0.94)** |
| ***Overweight or obesity*** |  |  |  |  |  |  |  |  |  |  |
| **No** | 19,604 | 8,664 | 88,032.79 | 98.42 | 24,497 | 8,119 | 53,068.56 | 152.99 | **0.62 (0.61, 0.64)** | **0.67 (0.52, 0.85)** |
| **Yes** | 53,574 | 16,357 | 271,261.30 | 60.30 | 55,560 | 11,539 | 132,426.06 | 87.14 | **0.67 (0.65, 0.69)** | **0.80 (0.73, 0.87)** |
| ***Polypharmacy*** |  |  |  |  |  |  |  |  |  |  |
| **1-4** | 13,262 | 2,456 | 71,963.55 | 34.13 | 14,364 | 1,697 | 35,568.00 | 47.71 | **0.68 (0.64, 0.73)** | **0.78 (0.63, 0.97)** |
| **5-9** | 36,434 | 11,316 | 185,145.57 | 61.12 | 38,186 | 8,309 | 91,922.53 | 90.39 | **0.65 (0.63, 0.67)** | **0.82 (0.76, 0.89)** |
| **≥10** | 23,482 | 11,249 | 102,184.97 | 110.08 | 27,507 | 9,652 | 58,003.09 | 166.40 | **0.64 (0.62, 0.65)** | **0.80 (0.74, 0.87)** |

Reference group: DOAC users

Abbreviations: CPRD = Clinical Research Practice Datalink, DOAC = direct oral anticoagulant, HR = hazard ratio, CI = confidence interval

**Table S8 Number of events, accumulated person-time, and unadjusted and propensity score weighted hazard ratios of all-cause mortality in warfarin and DOACs groups, CDARS - Subgroup analyses**

|  | **Warfarin users** | | | | **DOACs users** | | | | **Unadjusted HR (95% CI)** | **Propensity score weighted HR (95% CI)** |
| --- | --- | --- | --- | --- | --- | --- | --- | --- | --- | --- |
|  | **Number of persons** | **Number of events** | **Person-years at risk** | **Rate per 1,000** | **Number of persons** | **Number of events** | **Person-years at risk** | **Rate per 1,000** |  |  |
| ***Age*** |  |  |  |  |  |  |  |  |  |  |
| **18-<40** | 148 | 11 | 795.70 | 13.82 | 84 | 4 | 231.52 | 17.28 | 0.80 (0.26, 2.52) | 0.48 (0.12, 1.94) |
| **40-<50** | 427 | 51 | 2,165.66 | 23.55 | 345 | 21 | 1,081.87 | 19.41 | 1.21 (0.73, 2.01) | 1.61 (0.89, 2.92) |
| **50-<60** | 1,510 | 222 | 7,597.12 | 29.22 | 1,640 | 71 | 4,800.17 | 14.79 | **1.97 (1.51, 2.57)** | **1.76 (1.26, 2.45)** |
| **60-<70** | 3,117 | 530 | 13,922.67 | 38.07 | 5,180 | 328 | 13,940.42 | 23.53 | **1.62 (1.41, 1.86)** | **1.30 (1.09, 1.55)** |
| **70-<80** | 4,076 | 1,356 | 17,634.99 | 76.89 | 8,081 | 1,077 | 21,734.08 | 49.55 | **1.56 (1.44, 1.69)** | **1.31 (1.19, 1.45)** |
| **80+** | 3,790 | 2,017 | 11,844.43 | 170.29 | 9,903 | 2,434 | 20,483.44 | 118.83 | **1.45 (1.37, 1.54)** | **1.32 (1.22, 1.43)** |
| ***Sex*** |  |  |  |  |  |  |  |  |  |  |
| **Male** | 7,183 | 2,283 | 29,444.10 | 77.54 | 12,592 | 1,996 | 30,718.27 | 64.98 | **1.25 (1.17, 1.32)** | **1.30 (1.20, 1.40)** |
| **Female** | 5,885 | 1,904 | 24,516.47 | 77.66 | 12,641 | 1,939 | 31,553.22 | 61.45 | **1.32 (1.24, 1.40)** | **1.33 (1.22, 1.44)** |
| ***Calendar year of first OAC Rx date*** |  |  |  |  |  |  |  |  |  |  |
| **Before 2017** | 9,939 | 3,616 | 49,498.69 | 73.07 | 9,989 | 2,521 | 43,302.40 | 58.22 | **1.25 (1.19, 1.32)** | **1.22 (1.15, 1.30)** |
| **After 2017 (inclusive)** | 3,129 | 571 | 4,470.88 | 127.72 | 15,244 | 1,414 | 18,969.09 | 74.54 | **1.76 (1.59, 1.94)** | **1.58 (1.42, 1.77)** |
| ***Chronic kidney disease*** |  |  |  |  |  |  |  |  |  |  |
| **No** | 11,808 | 3,443 | 48,987.30 | 70.28 | 24,622 | 3,763 | 60,758.70 | 61.93 | **1.19 (1.13, 1.25)** | **1.30 (1.23, 1.38)** |
| **Yes** | 1,260 | 744 | 4,973.27 | 149.60 | 611 | 172 | 1,512.79 | 113.70 | **1.37 (1.16, 1.62)** | **1.65 (1.29, 2.11)** |
| ***Overweight or obesity*** |  |  |  |  |  |  |  |  |  |  |
| **No** | 10,518 | 3,234 | 43,358.03 | 74.59 | 19,886 | 3,062 | 48,969.77 | 62.53 | **1.25 (1.18, 1.31)** | **1.28 (1.20, 1.36)** |
| **Yes** | 2,550 | 953 | 10,602.55 | 89.88 | 5,347 | 873 | 13,301.72 | 65.63 | **1.42 (1.30, 1.56)** | **1.45 (1.28, 1.63)** |
| ***Polypharmacy*** |  |  |  |  |  |  |  |  |  |  |
| **0** | 2,203 | 422 | 4,030.06 | 104.71 | 9,433 | 1,003 | 14,200.32 | 70.63 | **1.51 (1.35, 1.69)** | **1.56 (1.37, 1.77)** |
| **1-4** | 2,011 | 430 | 11,593.13 | 37.09 | 2,579 | 244 | 8,756.27 | 27.87 | **1.32 (1.13, 1.54)** | 1.18 (0.96, 1.44) |
| **5-9** | 4,370 | 1,334 | 21,695.46 | 61.49 | 6,815 | 1,054 | 22,185.20 | 47.51 | **1.29 (1.19, 1.40)** | **1.32 (1.19, 1.46)** |
| **≥10** | 4,484 | 2,001 | 16,641.93 | 120.24 | 6,406 | 1,634 | 17,129.70 | 95.39 | **1.27 (1.19, 1.36)** | **1.31 (1.20, 1.43)** |

Reference group: DOAC users

Abbreviations: CDARS = Clinical Data Analysis and Reporting System, DOAC = direct oral anticoagulant, HR = hazard ratio, CI = confidence interval
