# Supplementary material for: Risk of mortality between warfarin and direct oral anticoagulants: population-based cohort studies
Source: BMC Med. 2024 Dec 23;22:597. doi: 10.1186/s12916-024-03808-y (PMC11664815; doi:10.1186/s12916-024-03808-y)
Supplement: Supplementary file 7 — Additional file 7: Table S9-16. Table S9. Number of events, accumulated person-time, and unadjusted and propensity score weighted hazard ratios of all-cause mortality in warfarin and DOACs groups, CPRD Aurum—Secondary analysis: warfarin TTR ≥ 65%, < 65% vs DOACs. Table S10. Number of events, accumulated person-time, and unadjusted and propensity score weighted hazard ratios of all-cause mortality in warfarin and DOACs groups, CDARS—Secondary analysis: warfarin TTR ≥ 65%, < 65% vs DOACs. Table S11. Number of events, accumulated person-time, and unadjusted and propensity score weighted hazard ratios of circulatory death in warfarin and DOACs groups, CPRD Aurum—Secondary post hoc analysis: warfarin TTR ≥ 65%, < 65% vs DOACs. Table S12. Number of events, accumulated person-time, and unadjusted and propensity score weighted hazard ratios of non-circulatory death in warfarin and DOACs groups, CPRD Aurum—Secondary post hoc analysis: warfarin TTR ≥ 65%, < 65% vs DOACs. Table S13. Number of events, accumulated person-time, and unadjusted and propensity score weighted hazard ratios of circulatory death in warfarin and DOACs groups, CDARS—Secondary post hoc analysis: warfarin TTR ≥ 65%, < 65% vs DOACs. Table S14. Number of events, accumulated person-time, and unadjusted and propensity score weighted hazard ratios of non-circulatory death in warfarin and DOACs groups, CDARS—Secondary post hoc analysis: warfarin TTR ≥ 65%, < 65% vs DOACs. Table S15 Number of events, accumulated person-time, and unadjusted and propensity score weighted hazard ratios of all-cause mortality in warfarin and DOACs groups, CPRD Aurum—Secondary analysis: warfarin vs individual DOACs. Table S16 Number of events, accumulated person-time, and unadjusted and propensity score weighted hazard ratios of all-cause mortality in warfarin and DOACs groups, CDARS—Secondary analysis: warfarin vs individual DOACs. [file 12916_2024_3808_MOESM7_ESM.docx]

**Additional file 7 Tables of secondary analyses**

**Table S9 Number of events, accumulated person-time, and unadjusted and propensity score weighted hazard ratios of all-cause mortality in warfarin and DOACs groups, CPRD Aurum - Secondary analysis: warfarin TTR≥65%, <65% vs DOACs**

|  | **Number of persons** | **Number of events** | **Person-years at risk** | **Rate per 1,000** | **Unadjusted HR (95% CI)** | **Propensity score weighted HR (95% CI)** |
| --- | --- | --- | --- | --- | --- | --- |
| **DOACs users** | 80,057 | 19,658 | 185,494.62 | 105.98 | 1.00 (Ref) | 1.00 (Ref) |
| **Warfarin TTR≥65% users** | 55,499 | 16,908 | 286,368.35 | 59.04 | **0.51 (0.50, 0.52)** | **0.68 (0.65, 0.72)** |
| **Warfarin TTR<65% users** | 6,471 | 2,889 | 27,047.62 | 106.81 | 1.04 (1.00, 1.08) | **1.14 (1.05, 1.23)** |

Abbreviations: CPRD = Clinical Research Practice Datalink, DOAC = direct oral anticoagulant, HR = hazard ratio, CI = confidence interval, Ref = reference group. TTR = Time in Therapeutic Range

**Table S10 Number of events, accumulated person-time, and unadjusted and propensity score weighted hazard ratios of all-cause mortality in warfarin and DOACs groups, CDARS - Secondary analysis: warfarin TTR≥65%, <65% vs DOACs**

|  | **Number of persons** | **Number of events** | **Person-years at risk** | **Rate per 1,000** | **Unadjusted HR**  **(95% CI)** | **Propensity score weighted HR**  **(95% CI)** |
| --- | --- | --- | --- | --- | --- | --- |
| **DOACs users** | 25,233 | 3,935 | 62,271.49 | 63.19 | 1.00 (Ref) | 1.00 (Ref) |
| **Warfarin TTR≥65% users** | 4,788 | 1,036 | 24,507.38 | 42.27 | **0.65 (0.61, 0.70)** | **0.86 (0.77, 0.96)** |
| **Warfarin TTR<65% users** | 8,113 | 3,139 | 28,776.56 | 109.08 | **1.78 (1.70, 1.87)** | **1.59 (1.50, 1.69)** |

Abbreviations: CDARS = Clinical Data Analysis and Reporting System, DOAC = direct oral anticoagulant, HR = hazard ratio, CI = confidence interval, Ref = reference group. TTR = Time in Therapeutic Range

**Table S11 Number of events, accumulated person-time, and unadjusted and propensity score weighted hazard ratios of circulatory death in warfarin and DOACs groups, CPRD Aurum - Secondary post hoc analysis: warfarin TTR≥65%, <65% vs DOACs**

|  | **Number of persons** | **Number of events** | **Person-years at risk** | **Rate per 1,000** | **Unadjusted HR**  **(95% CI)** | **Propensity score weighted HR**  **(95% CI)** |
| --- | --- | --- | --- | --- | --- | --- |
| **DOACs users** | 80,057 | 7,421 | 185,461.12 | 40.01 | 1.00 (Ref) | 1.00 (Ref) |
| **Warfarin TTR≥65% users** | 55,499 | 6,340 | 286,339.42 | 22.14 | **0.52 (0.50, 0.54)** | **0.69 (0.64, 0.74)** |
| **Warfarin TTR<65% users** | 6,471 | 1,055 | 27,042.60 | 39.01 | 1.03 (0.96, 1.10) | 1.05 (0.92, 1.20) |

Abbreviations: CPRD = Clinical Research Practice Datalink, DOAC = direct oral anticoagulant, HR = hazard ratio, CI = confidence interval, Ref = reference group. TTR = Time in Therapeutic Range

**Table S12 Number of events, accumulated person-time, and unadjusted and propensity score weighted hazard ratios of non-circulatory death in warfarin and DOACs groups, CPRD Aurum - Secondary post hoc analysis: warfarin TTR≥65%, <65% vs DOACs**

|  | **Number of persons** | **Number of events** | **Person-years at risk** | **Rate per 1,000** | **Unadjusted HR**  **(95% CI)** | **Propensity score weighted HR**  **(95% CI)** |
| --- | --- | --- | --- | --- | --- | --- |
| **DOACs users** | 80,057 | 12,237 | 185,474.30 | 65.98 | 1.00 (Ref) | 1.00 (Ref) |
| **Warfarin TTR≥65% users** | 55,499 | 10,568 | 286,351.00 | 36.91 | **0.50 (0.49, 0.52)** | **0.68 (0.63, 0.73)** |
| **Warfarin TTR<65% users** | 6,471 | 1,834 | 27,044.73 | 67.81 | 1.05 (1.00, 1.10) | **1.19 (1.07, 1.31)** |

Abbreviations: CPRD = Clinical Research Practice Datalink, DOAC = direct oral anticoagulant, HR = hazard ratio, CI = confidence interval, Ref = reference group. TTR = Time in Therapeutic Range

**Table S13 Number of events, accumulated person-time, and unadjusted and propensity score weighted hazard ratios of circulatory death in warfarin and DOACs groups, CDARS - Secondary post hoc analysis: warfarin TTR≥65%, <65% vs DOACs**

|  | **Number of persons** | **Number of events** | **Person-years at risk** | **Rate per 1,000** | **Unadjusted HR**  **(95% CI)** | **Propensity score weighted HR**  **(95% CI)** |
| --- | --- | --- | --- | --- | --- | --- |
| **DOACs users** | 25,233 | 994 | 62,263.44 | 15.96 | 1.00 (Ref) | 1.00 (Ref) |
| **Warfarin TTR≥65% users** | 4,788 | 378 | 24,505.58 | 15.43 | 0.97 (0.86, 1.10) | 1.09 (0.90, 1.32) |
| **Warfarin TTR<65% users** | 8,113 | 904 | 28,770.44 | 31.42 | **2.09 (1.91, 2.29)** | **1.80 (1.61, 2.01)** |

Abbreviations: CDARS = Clinical Data Analysis and Reporting System, DOAC = direct oral anticoagulant, HR = hazard ratio, CI = confidence interval, Ref = reference group. TTR = Time in Therapeutic Range

**Table S14 Number of events, accumulated person-time, and unadjusted and propensity score weighted hazard ratios of non-circulatory death in warfarin and DOACs groups, CDARS - Secondary post hoc analysis: warfarin TTR≥65%, <65% vs DOACs**

|  | **Number of persons** | **Number of events** | **Person-years at risk** | **Rate per 1,000** | **Unadjusted HR**  **(95% CI)** | **Propensity score weighted HR**  **(95% CI)** |
| --- | --- | --- | --- | --- | --- | --- |
| **DOACs users** | 25,233 | 2,941 | 62,268.77 | 47.23 | 1.00 (Ref) | 1.00 (Ref) |
| **Warfarin TTR≥65% users** | 4,788 | 658 | 24,506.35 | 26.85 | **0.55 (0.50, 0.60)** | **0.78 (0.68, 0.89)** |
| **Warfarin TTR<65% users** | 8,113 | 2,235 | 28,774.09 | 77.67 | **1.68 (1.59, 1.78)** | **1.53 (1.43, 1.64)** |

Abbreviations: CDARS = Clinical Data Analysis and Reporting System, DOAC = direct oral anticoagulant, HR = hazard ratio, CI = confidence interval, Ref = reference group. TTR = Time in Therapeutic Range

**Table S15 Number of events, accumulated person-time, and unadjusted and propensity score weighted hazard ratios of all-cause mortality in warfarin and DOACs groups, CPRD Aurum - Secondary analysis: warfarin vs individual DOACs**

|  | **Number of persons** | **Number of events** | **Person-years at risk** | **Rate per 1,000** | **Unadjusted HR**  **(95% CI)** | **Propensity score weighted HR**  **(95% CI)** |
| --- | --- | --- | --- | --- | --- | --- |
| **Warfarin users** | 73,178 | 25,021 | 359,294.09 | 69.64 | 1.00 (Ref) | 1.00 (Ref) |
| **Edoxaban users** | 3,857 | 427 | 4,276.18 | 99.86 | **1.51 (1.37, 1.67)** | **0.15 (0.03, 0.76)** |
| **Rivaroxaban users** | 32,049 | 8,686 | 84,663.04 | 102.59 | **1.53 (1.49, 1.57)** | **1.22 (1.05, 1.41)** |
| **Dabigatran users** | 5,922 | 1,824 | 21,485.61 | 84.89 | **1.24 (1.18, 1.30)** | **1.30 (1.16, 1.44)** |
| **Apixaban users** | 38,240 | 8,727 | 73,744.07 | 118.34 | **1.76 (1.72, 1.81)** | 0.89 (0.53, 1.49) |

Abbreviations: CPRD = Clinical Research Practice Datalink, DOAC = direct oral anticoagulant, HR = hazard ratio, CI = confidence interval, Ref = reference group. TTR = Time in Therapeutic Range

**Table S16 Number of events, accumulated person-time, and unadjusted and propensity score weighted hazard ratios of all-cause mortality in warfarin and DOACs groups, CDARS - Secondary analysis: warfarin vs individual DOACs**

|  | **Number of persons** | **Number of events** | **Person-years at risk** | **Rate per 1,000** | **Unadjusted HR**  **(95% CI)** | **Propensity score weighted HR**  **(95% CI)** |
| --- | --- | --- | --- | --- | --- | --- |
| **Warfarin users** | 13,068 | 4,187 | 53,960.57 | 77.59 | 1.00 (Ref) | 1.00 (Ref) |
| **Apixaban users** | 8,700 | 1,117 | 12,991.42 | 85.98 | 0.94 (0.88, 1.01) | **0.57 (0.38, 0.86)** |
| **Edoxaban users** | 478 | 41 | 447.12 | 91.70 | 0.85 (0.62, 1.16) | **0.58 (0.36, 0.95)** |
| **Rivaroxaban users** | 5,392 | 955 | 16,049.89 | 59.50 | **0.74 (0.69, 0.80)** | **0.62 (0.44, 0.87)** |
| **Dabigatran users** | 10,659 | 1,821 | 32,660.95 | 55.75 | **0.70 (0.67, 0.74)** | **0.82 (0.76, 0.88)** |

Abbreviations: CDARS = Clinical Data Analysis and Reporting System, DOAC = direct oral anticoagulant, HR = hazard ratio, CI = confidence interval, Ref = reference group. TTR = Time in Therapeutic Range
