# Supplementary material for: Risk of mortality between warfarin and direct oral anticoagulants: population-based cohort studies
Source: BMC Med. 2024 Dec 23;22:597. doi: 10.1186/s12916-024-03808-y (PMC11664815; doi:10.1186/s12916-024-03808-y)
Supplement: Supplementary file 8 — Additional file 8: Table S17. Table S17. Sensitivity analyses. [file 12916_2024_3808_MOESM8_ESM.docx]

**Additional file 8 Tables of sensitivity analyses**

**Table S17 Sensitivity analyses**

|  | **CPRD Aurum**  **Original propensity score weighted estimate**  **HR 0.81, 95% CI (0.77, 0.86)** | **CDARS**  **Original propensity score weighted estimate**  **HR 1.31, 95% CI (1.24, 1.39)** |
| --- | --- | --- |
|  | **Adjusted/Propensity score weighted estimate**  **HR (95% CI)** | **Adjusted/Propensity score weighted estimate**  **HR (95% CI)** |
| **Multivariable regression model** | 0.75 (0.73, 0.77) | 1.36 (1.29, 1.43) |
| **Trimming PSs <5% strata*** | 0.83 (0.78, 0.88) | 1.25 (1.18, 1.33) |
| **Including topical medication in polypharmacy** | 0.81 (0.77, 0.86) | 1.31 (1.24, 1.39) |
| **Applying multiple imputation** | 0.80 (0.76, 0.85) | NA |
| **Restricting study period to 2014-2019** | 0.75 (0.73, 0.78) | 1.42 (1.33, 1.51) |
| **Only using covariates available in both CPRD and CDARS for propensity score weighting** | 0.80 (0.76, 0.85) | 1.32 (1.25, 1.39) |
| **Censoring discontinuation** | 0.52 (0.49, 0.56) | 1.23 (1.14, 1.32) |

Reference group: DOAC users

Abbreviations: CPRD = Clinical Research Practice Datalink, CDARS = Clinical Data Analysis and Reporting System, DOAC = direct oral anticoagulant, HR = hazard ratio, CI = confidence interval, PS = propensity score, NA = not applicable

*Strata 1-4 were trimmed in CPRD Aurum; Strata 1-6 were trimmed in CDARS
