# Supplementary material for: Risk of mortality between warfarin and direct oral anticoagulants: population-based cohort studies
Source: BMC Med. 2024 Dec 23;22:597. doi: 10.1186/s12916-024-03808-y (PMC11664815; doi:10.1186/s12916-024-03808-y)
Supplement: Supplementary file 9 — Additional file 9: Figure S1-2.Figure S1. E-value for the lower 95% confidence interval and point estimate in atrial fibrillation with anticoagulant use in CPRD Aurum. Figure S2. E-value for the lower 95% confidence interval and point estimate in atrial fibrillation with anticoagulant use in CDARS. [file 12916_2024_3808_MOESM9_ESM.docx]

**Additional file 9 Quantitative bias analyses**

**Figure S1 E-value for the lower 95% confidence interval and point estimate in atrial fibrillation with anticoagulant use in CPRD Aurum**

**
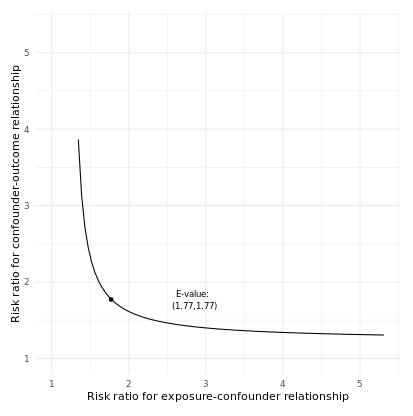
**

Abbreviations: CPRD = Clinical Research Practice Datalink

**Figure S2 E-value for the lower 95% confidence interval and point estimate in atrial fibrillation with anticoagulant use in CDARS**

**
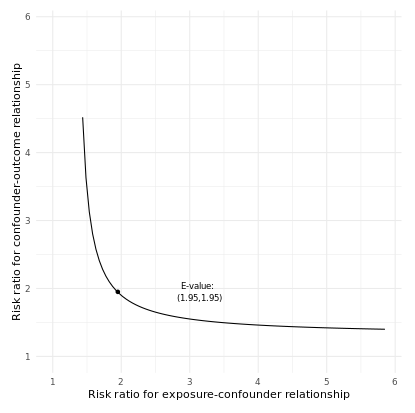
**

Abbreviations: CDARS = Clinical Data Analysis and Reporting System
